# The difference of paraspinal muscle between patients with lumbar spinal stenosis and normal middle-aged and elderly people, studying by propensity score matching

**DOI:** 10.3389/fendo.2022.1080033

**Published:** 2022-11-22

**Authors:** Wei Wang, Yang Guo, Weishi Li, Zhongqiang Chen

**Affiliations:** ^1^ Department of Orthopaedics, Tianjin Hospital, Tianjin, China; ^2^ Department of Orthopaedics, Peking University Third Hospital, Beijing, China; ^3^ Beijing Key Laboratory of Spinal Disease Research, Beijing, China; ^4^ Engineering Research Center of Bone and Joint Precision Medicine, Ministry of Education, Beijing, China

**Keywords:** multifidus, erector spinae muscle, paraspinal muscle degeneration, normal people, lumbar spinal stenosis

## Abstract

**Objective:**

The purpose of this study was to elaborate the characteristics of paraspinal muscles in lower lumbar, to compare the differences of paraspinal muscle between patients with lumbar spinal stenosis and normal people and to explore the influencing factors of paraspinal muscle degeneration in patients with lumbar spinal stenosis.

**Method:**

The 39 pairs of patients and normal people were selected by propensity score matching. The differences of multifidus muscle and erection spine muscle parameters between the two groups were compared by independent-samples t-test and the relationship between age, paraspinal muscle degeneration and other factors in patients with lumbar spinal stenosis was analyzed by Pearson or Spearman correlation analysis.

**Result:**

The general conditions of the two groups (patients with lumbar spinal stenosis and normal people) were well matched. There were significant differences in the relative fatty cross sectional area, fatty infiltration and relative signal intensity of multifidus muscle at L3 level. The fatty infiltration and relative signal intensity of multifidus muscle at L4 level and the relative signal intensity of multifidus muscle at L5 level were also significantly different. For male, the relative fatty cross sectional area, the fatty infiltration and relative signal intensity of multifidus muscle in patients were higher than those in healthy peers. For female, the relative signal intensity of multifidus muscle in patients was higher, too. In patients group, age was significantly correlated with the relative fatty cross sectional area, fatty infiltration and relative signal intensity of multifidus muscle and erector spinae muscle. Weight and BMI were significantly correlated with the relative total cross-sectional area of erector spinae muscle. The fatty infiltration increased more significantly with age in patients than that in normal people.

**Conclusion:**

The change rules of paraspinal muscles in patients with lumbar spinal stenosis are similar to those in normal people. The degeneration of paraspinal muscle in patients with lumbar spinal stenosis was more severe than that in normal people, mostly in multifidus muscle. The paraspinal muscle degeneration was related to age in patients, and the effect of age on atrophy of paraspinal muscle was greater than that of normal people.

## Introduction

With the aging of the population, the incidence of lumbar degenerative diseases is gradually increasing. Recently, many studies focused on the degeneration of paraspinal muscle in lumbar degenerative diseases (1–6), because paraspinal muscle plays an important role in maintaining stability. Ogon studied the degeneration of paraspinal muscles in 40 pairs of patients with chronic nonspecific low back pain and lumbar spinal stenosis (7). Other studies also investigated the degeneration of paraspinal muscle in patients with low back pain (8, 9) and lumbar degenerative kyphosis (3, 10).

Lumbar spinal stenosis (LSS) is one of the most common lumbar degenerative diseases (11), which is associated with high social and economic burden (12). Many researches also explored the degeneration of paraspinal muscle in patients with LSS (5, 13, 14). Yagi investigated the degeneration of paraspinal muscle in patients with both simple LSS and degenerative scoliosis combined with LSS. By analyzing the data of 60 pairs of female patients, they found that the cross-sectional area of multifidus muscle was significantly smaller in patients with degenerative scoliosis combined with LSS than that in patients with simple LSS (15). Another study found that the decrease of cross-sectional area and atrophy in multifidus muscle was associated with poorer outcome in patients with LSS (16), while it only measured the parameters of multifidus muscle. Although these researches investigated the degeneration of paraspinal muscle in patients with LSS, the difference of paraspinal muscle between normal people and patients with lumbar spinal stenosis was unclear.

Previous studies focused on the degeneration of paraspinal muscles, especially in lower lumbar, while their results were not comparable due to the differences of measurements (17–20). Some studies measured paraspinal muscle parameters at single level of lumbar (13, 14, 21), while others measured at multi-levels (4, 10, 17). The parameters they measured were also different from each other. There lacked a study detailing paraspinal muscle parameters.

So the purpose of this study was to elaborate the characteristics of paraspinal muscles in lower lumbar, to compare the differences of paraspinal muscle between patients with LSS and normal people and to explore the influencing factors of paraspinal muscle degeneration in patients with LSS.

## Method

### General information

This study was approved by the Ethics Committee of Peking University Third Hospital. There were 93 patients in our study, who were diagnosed as lumbar spinal stenosis and underwent posterior lumbar decompression and fusion surgery from October 2018 to June 2019. The inclusion criteria were (i) age was from 50 to 80 years old, (ii) diagnosed as lumbar spinal stenosis, (iii) undertook lumbar MRI test. The exclusion criteria were (i) with other spinal diseases, (ii) with a history of spinal surgery, (iii) with neuromuscular diseases, (iiii) lumbar MRI was uncomplete. Control group included 45 normal middle-aged and elderly people, who were prospectively recruited from February 2020 to November 2020. The inclusion criteria were (i) age was from 50 to 80 years old, (ii) without spinal diseases, (iii) without spinal surgery, (iiii) without low back pain and trauma in past 3 months. The exclusion criteria were (i) with neuromuscular diseases, (ii) with MRI contraindications. All the normal people signed the informed consent forms.

### Clinical measurements

The data of both patients and normal people was recorded, including the age, gender, height, weight and history of hypertension or diabetes. All patients underwent lumbar magnetic resonance imaging (MRI) within 1 month before surgery. All normal people underwent lumbar MRI within 1 month before we measured the parameters.

Measurements of the multifidus (MF) and erector spinae muscle (ES) were obtained from T2-weighted images by Image J software. MRIs were required with Signa HDxt 3.0T (General Electric Company, USA). Patients were placed in the supine position, with their legs straight and the lumbar spine in a neutral posture. Axial MRI was parallel to the inferior endplate of the vertebral body. All muscles were measured bilaterally at the inferior vertebral endplate of L3 to L5. The mean value of left and right paraspinal muscle was calculated. Region of interest was used to measure muscular parameters, including: total cross-sectional area (tCSA), fatty cross-sectional area (fCSA), fatty infiltration (FI) and signal intensity (SI). The fCSA was defined as the area of fatty tissue in muscle, which was measured by the thresholding technique. The FI was defined as the ratio of fCSA to tCSA. They reflected the degeneration of paraspinal muscles.

In order to reduce the influence of height, weight and body size on paraspinal muscle parameters, we calculated the relative cross-sectional area (rCSA) and relative signal intensity (rSI). The relative total cross-sectional area (rtCSA) was defined as the ratio of cross-sectional area of paraspinal muscle to cross-sectional area of vertebral body. The relative fatty cross-sectional area (rfCSA) was defined as the ratio of cross-sectional area of fatty tissue to cross-sectional area of vertebral body. The relative signal intensity (rSI) was defined as the ratio of signal intensity of paraspinal muscle to signal intensity of subcutaneous fat.

### Statistical analysis

SPSS version 22.0 (IBM company, USA) was used to analyze the collected data. By using propensity score matching, we matched 93 patients with LSS and 45 normal people at a ratio of 1:1. The matching model we used was logistic regression model. To get a good matching score, the influencing factors such as age, gender, height, weight and body mass index (BMI) were used as matching indexes. The values were expressed as mean ± standard deviation. Age, BMI and paraspinal muscle parameters were continuous variable while gender was categorical variable. We measured the paraspinal muscle parameters at a level of L3 to L5 and analyzed the change rule of paraspinal muscle parameters. The differences of paraspinal muscle parameters between patients with LSS and normal people were compared. The independent sample t-test or rank sum test were used to explore the difference of continuous variables between the two groups, while the chi-square test was used to analyze the difference of categorical variables. Correlations between measurements of paraspinal muscle and other factors were investigated by Pearson correlation analysis or Spearman correlation analysis. Statistical significance was set at p-value < 0.05.

## Results

By using propensity score matching, we matched 39 pairs of patients with LSS and normal middle-age and elderly people well. As showed in **Table 1**, there were 18 males and 21 females in patients group. The average age of patients was 62.9 ± 7.8 years. 10 patients were diagnosed as hypertension and 4 patients were diagnosed as diabetes. In normal middle-age and elderly people group (normal group), there were 16 males and 23 females. The average age of normal people was 62.1 ± 7.3 years. 10 patients were diagnosed as hypertension and 6 patients were diagnosed as diabetes. There was no significant difference in age, gender, height, weight, BMI and comorbidities between the two groups, indicating that the two groups were matched well.

**Table 1:** The basic information of two groups.

Parameters	Patients group	Normal group	P-value
**Age (years)**	62.9 ± 7.8	62.1 ± 7.3	0.666
**Gender (M/F)**	18/21	16/23	0.648
**Height (cm)**	163.7 ± 8.7	163.2 ± 9.1	0.767
**Weight (kg)**	68.2 ± 10.3	66.1 ± 11.8	0.502
**BMI (kg/m^2^)**	25.4 ± 2.9	24.7 ± 2.9	0.298
**Hypertension (Y/N)**	10/29	10/29	1.00
**Diabetes (Y/N)**	4/35	6/33	0.735

BMI, body mass index.

To explore the differences in paraspinal muscles between patients with LSS and normal middle-aged and elderly people, we measured the praspinal muscle parameters, including rtCSA, rfCSA, FI and rSI. The results were recorded in **Table 2**. Compared with the normal group, the change rules of paraspinal muscle in the patient group were similar. From top to bottom of the spinal axis, the relative cross-sectional area of MF increases, while that of ES decreases. The FI and rSI of MF and ES increased gradually.

**Table 2:** The comparison of paraspinal muscle parameters at L3 to L5 levels.

Parameters	Patients group	Normal group	P-value
**L3**			
**MF rtCSA**	0.96 ± 0.30	0.94 ± 0.17	0.731
**MF rfCSA**	0.32 ± 0.17	0.27 ± 0.15	0.042*
**ES rtCSA**	2.60 ± 0.62	2.71 ± 0.43	0.385
**ES rfCSA**	0.56 ± 0.24	0.59 ± 0.22	0.415
**MF FI**	0.34 ± 0.14	0.28 ± 0.11	0.013*
**ES FI**	0.22 ± 0.08	0.22 ± 0.08	0.948
**MF rSI**	0.49 ± 0.13	0.40 ± 0.09	<0.01**
**ES rSI**	0.41 ± 0.09	0.37 ± 0.08	0.030*
**L4**			
**MF rtCSA**	1.29 ± 0.32	1.29 ± 0.24	0.946
**MF rfCSA**	0.48 ± 0.23	0.41 ± 0.19	0.062
**ES rtCSA**	2.20 ± 0.52	2.38 ± 0.40	0.083
**ES rfCSA**	0.66 ± 0.29	0.71 ± 0.23	0.412
**MF FI**	0.37 ± 0.15	0.31 ± 0.11	0.044*
**ES FI**	0.29 ± 0.10	0.30 ± 0.09	0.964
**MF rSI**	0.53 ± 0.13	0.44 ± 0.11	0.002**
**ES rSI**	0.47 ± 0.11	0.43 ± 0.10	0.116
**L5**			
**MF rtCSA**	1.52 ± 0.36	1.55 ± 0.32	0.630
**MF rfCSA**	0.55 ± 0.20	0.50 ± 0.23	0.102
**ES rtCSA**	1.39 ± 0.41	1.55 ± 0.42	0.088
**ES rfCSA**	0.57 ± 0.26	0.63 ± 0.25	0.143
**MF FI**	0.37 ± 0.15	0.32 ± 0.11	0.087
**ES FI**	0.42 ± 0.14	0.40 ± 0.12	0.653
**MF rSI**	0.54 ± 0.13	0.47 ± 0.11	0.014*
**ES rSI**	0.56 ± 0.11	0.51 ± 0.10	0.070

MF, multifidus; ES, erector spinae; FI, fatty infiltration; rtCSA, relative total cross sectional area; rfCSA, relative fatty cross sectional area; rSI, relative signal intensity *means P value < 0.05; **means P value < 0.01.

For paraspinal muscle parameters at L3 level, there were significant differences in the rfCSA, FI, rSI of MF and rSI of ES (P<0.05). The FI and rSI of MF at L4 level and the rSI of MF at L5 level were also significantly different (P<0.05). Although there was no significant difference in rfCSA of MF and rSI of ES at L4 level and rfCSA, FI of MF, and rSI of ES at L5 level, those parameters of patients were higher than those of normal people. The results reflected that the overall degeneration of paraspinal muscle in patients with LSS was worse than that in normal people.

**Figure 1:**
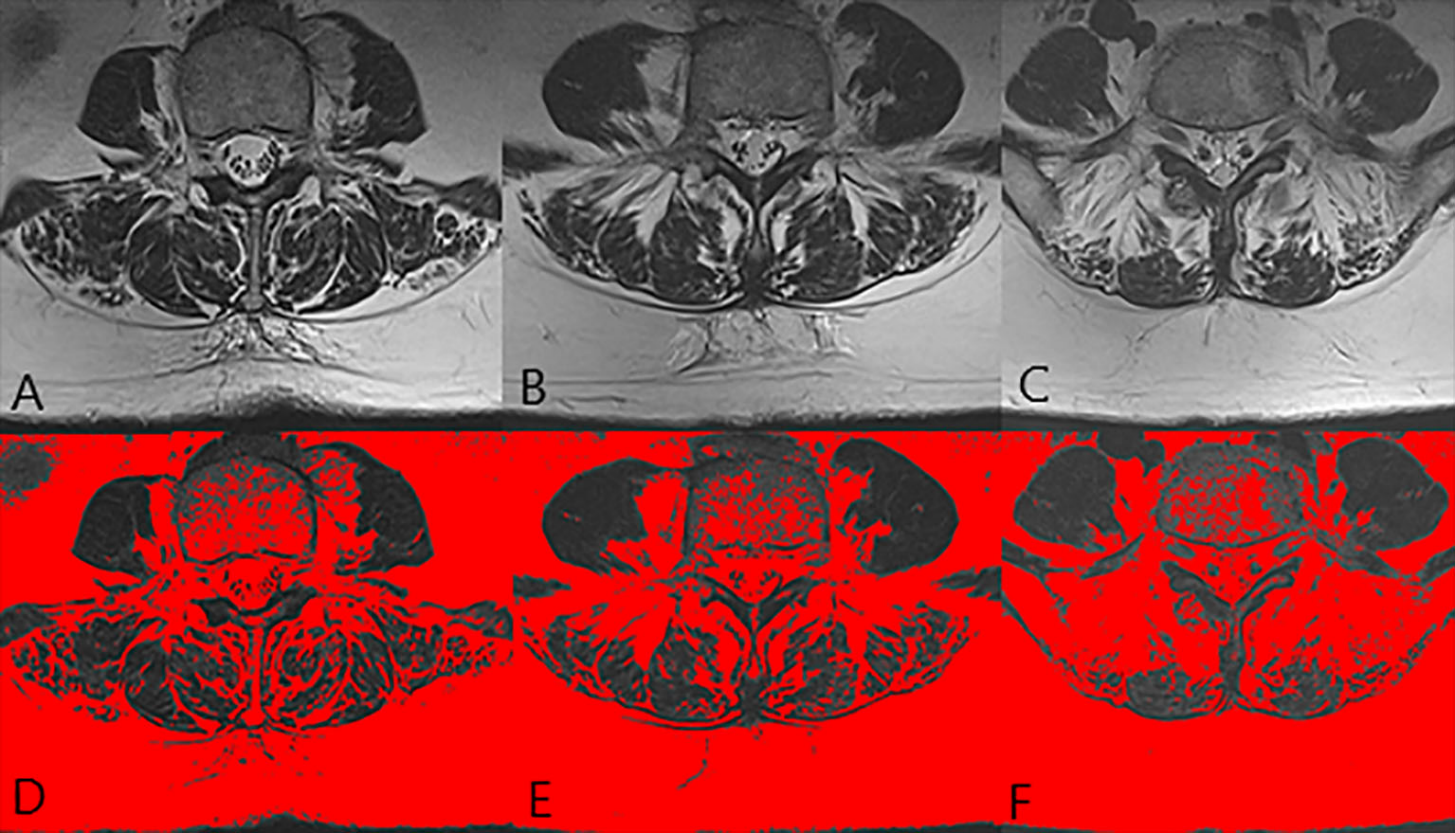
The images of paraspinal muscle in a 71 years old normal female. **(A, D)** were at L3 level. **(B, E)** were at L4 level. **(C, F)** were at L5 level. The FI of MF was 36.8% and the FI of ES was 48.2%.

**Figure 2:**
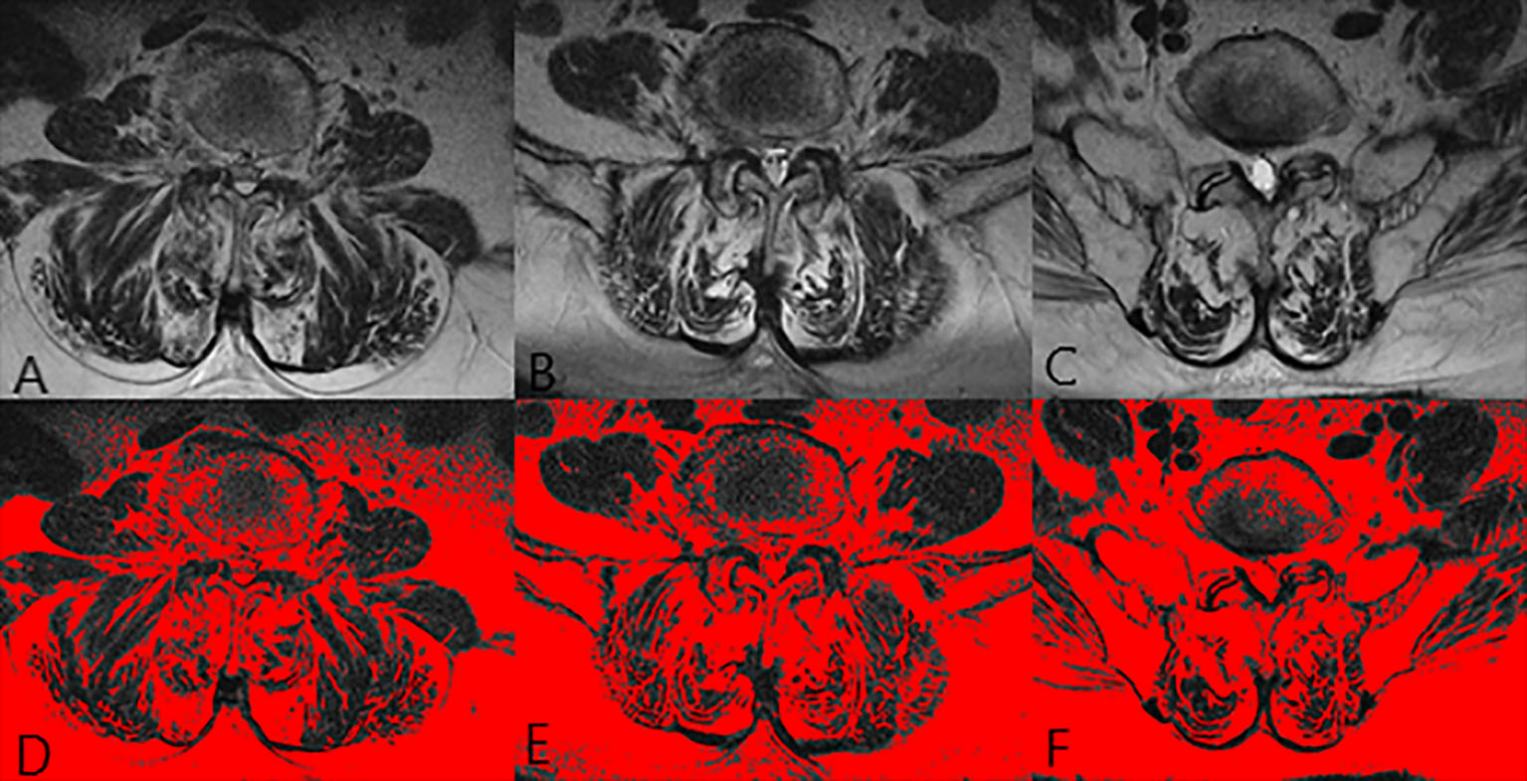
The images of paraspinal muscle in a 70 years old female patient. **(A, D)** were at L3 level. **(B, E)** were at L4 level. **(C, F)** were at L5 level. The FI of MF was 57.6% and the FI of ES was 43.0%.

We further compared the mean values of paraspinal muscle parameters from L3 to L5 level between the two groups (The mean value was used in the following analyses) and found that the rfCSA, FI, rSI of MF and rSI of ES in patients group were significantly higher than those in normal group (**Table 3**). The degeneration of paraspinal muscle in patients with LSS was significantly severer than that in normal people, which was mainly manifested in multifidus muscle (**Figures 1**, **2**).

**Table 3:** The mean values of paraspinal muscle parameters.

Parameters	Patients group	Normal group	P-value
MF rtCSA	1.25 ± 0.28	1.26 ± 0.22	0.897
MF rfCSA	0.45 ± 0.18	0.39 ± 0.18	0.013*
ES rtCSA	2.06 ± 0.38	2.21 ± 0.35	0.072
ES rfCSA	0.60 ± 0.20	0.64 ± 0.21	0.404
MF FI	0.36 ± 0.14	0.30 ± 0.10	0.014*
ES FI	0.31 ± 0.09	0.31 ± 0.09	0.671
MF rSI	0.52 ± 0.12	0.44 ± 0.10	0.001**
ES rSI	0.48 ± 0.09	0.44 ± 0.09	0.038*

MF, multifidus; ES, erector spinae; FI, fatty infiltration; rtCSA, relative total cross sectional area; rfCSA, relative fatty cross sectional area; rSI, relative signal intensity *means P value < 0.05; **means P value < 0.01.

These patients were divided into two groups according to the gender. There was no significant difference in paraspinal muscle parameters between the two groups. But compared with males, the rfCSA and FI of MF and ES were higher in females (**Table 4**).

**Table 4:** Comparison of paraspinal muscle parameters between genders in patients.

Parameter	Male	Female	P-value
**Age (yrs)**	62.2 ± 8.0	63.5 ± 7.9	0.610
**Height (cm)**	170.1 ± 7.3	158.3 ± 5.7	<0.01**
**Weight (kg)**	72.6 ± 10.0	64.4 ± 9.1	0.011*
**BMI (kg/m^2^)**	25.0 ± 2.6	25.7 ± 3.1	0.568
**MF rtCSA**	1.22 ± 0.30	1.29 ± 0.27	0.452
**MF rfCSA**	0.43 ± 0.16	0.47 ± 0.19	0.477
**ES rtCSA**	2.16 ± 0.38	1.98 ± 0.36	0.122
**ES rfCSA**	0.59 ± 0.18	0.61 ± 0.21	0.786
**MF FI**	0.36 ± 0.16	0.37 ± 0.12	0.832
**ES FI**	0.30 ± 0.11	0.32 ± 0.08	0.692
**MF rSI**	0.52 ± 0.15	0.52 ± 0.09	0.957
**ES rSI**	0.48 ± 0.10	0.48 ± 0.07	0.796

BMI, body mass index; MF, multifidus; ES, erector spinae; FI, fatty infiltration; rtCSA, relative total cross sectional area; rfCSA, relative fatty cross sectional area; rSI, relative signal intensity *means P value < 0.05; **means P value < 0.01.

Then we compared the differences of paraspinal muscle parameters between patients and normal people under different gender. As the results showed in **Table 5**, for males, the rfCSA, FI and rSI of MF were higher in patients than those in normal peers (p<0.05). For females, the rSI of MF was higher in patients than that in normal peers (p<0.05). Although there was no significant difference in the rfCSA and FI of MF between female patients and normal peers, the rfCSA and FI of MF were also higher in female patients.

**Table 5:** Comparison of paraspinal muscle parameters between patients and normal peers under different gender.

Parameter	Male			Female		
	Patients	Normal peers	P-value	Patients	Normal peers	P-value
Age (yrs)	62.2 ± 8.0	60.8 ± 7.2	0.592	63.5 ± 7.9	63.1 ± 7.4	0.991
Height (cm)	170.1 ± 7.3	171.6 ± 5.7	0.508	158.3 ± 5.7	157.4 ± 5.8	0.579
Weight (kg)	72.6 ± 10.0	74.6 ± 10.7	0.577	64.4 ± 9.1	60.3 ± 8.5	0.128
BMI(kg/m^2^)	25.0 ± 2.6	25.2 ± 2.5	0.817	25.7 ± 3.1	24.3 ± 3.1	0.154
MF rtCSA	1.22 ± 0.30	1.25 ± 0.21	0.721	1.29 ± 0.27	1.27 ± 0.23	0.835
MF rfCSA	0.43 ± 0.16	0.32 ± 0.09	0.020*	0.47 ± 0.19	0.44 ± 0.21	0.184
ES rtCSA	2.16 ± 0.38	2.31 ± 0.41	0.273	1.98 ± 0.36	2.14 ± 0.30	0.098
ES rfCSA	0.59 ± 0.18	0.56 ± 0.12	0.623	0.61 ± 0.21	0.70 ± 0.24	0.192
MF FI	0.36 ± 0.16	0.25 ± 0.05	0.025*	0.37 ± 0.12	0.34 ± 0.12	0.275
ES FI	0.30 ± 0.11	0.27 ± 0.06	0.198	0.32 ± 0.08	0.34 ± 0.10	0.613
MF rSI	0.52 ± 0.15	0.40 ± 0.10	0.009**	0.52 ± 0.09	0.46 ± 0.10	0.045*
ES rSI	0.48 ± 0.10	0.41 ± 0.09	0.059	0.48 ± 0.07	0.46 ± 0.09	0.273

BMI, body mass index; MF, multifidus; ES, erector spinae; FI, fatty infiltration; rtCSA, relative total cross sectional area; rfCSA, relative fatty cross sectional area; rSI, relative signal intensity *means P value < 0.05; **means P value < 0.01.

We used correlation analysis to explore the relationship between paraspinal muscle parameters and other factors, such as age, height, weight and BMI in patients with LSS and the results were recorded in **Table 6**. Age was significantly correlated with the rfCSA, FI and rSI of both MF and ES (p<0.05). Weight and BMI were significantly correlated with the rtCSA of ES (p<0.05).

**Table 6:** Relationships between paraspinal muscle parameters and other factors in patients.

Parameter	MF rtCSA	MF rfCSA	ES rtCSA	ES rfCSA	MF FI	ES FI	MF rSI	ES rSI
**Age**	-0.056	0.427**	-0.053	0.354*	0.499**	0.415**	0.498**	0.390*
**Height**	-0.100	-0.195	0.183	-0.100	-0.181	-0.216	-0.217	-0.228
**Weight**	0.150	0.094	0.378*	0.055	-0.148	-0.108	-0.171	-0.122
**BMI**	0.246	0.283	0.364*	0.289	0.038	0.145	0.077	0.118

BMI, body mass index; MF, multifidus; ES, erector spinae; FI, fatty infiltration; rtCSA, relative total cross sectional area; rfCSA, relative fatty cross sectional area; rSI, relative signal intensity *means P value < 0.05; **means P value < 0.01.

**Figure 3:**
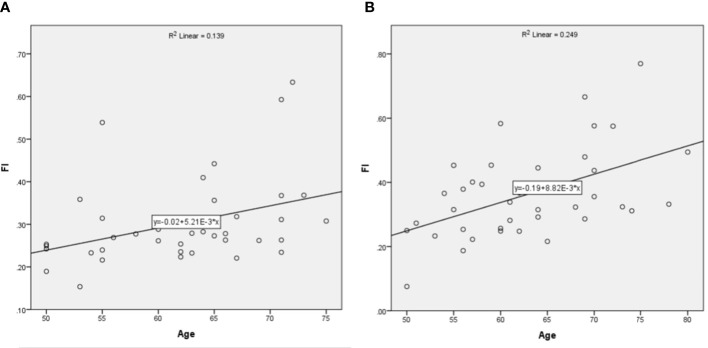
The relationship between age and FI of MF. **(A)** was in normal people and **(B)** was in patients.

The linear regression analysis was used to evaluate the relationship between age and the FI of MF. As showed in **Figure 3**, the slope of fitted linear was higher in patients than that in normal people, indicating that with the increase of age, the fatty infiltration increased more significantly in patients than that in normal people.

## Discussion

With the aging of population, the incidence of lumbar degenerative diseases is gradually increasing. Lumbar spinal stenosis is one of the common lumbar diseases and many researches focused on the degeneration of paraspinal muscle in lumbar spinal stenosis. Zotti found that the decrease of cross-sectional area of MF was correlated to the outcome in patients with LSS (16). But this study only measured the MF and lacked the data of normal people. In this study, we found that the degeneration of paraspinal muscle was worse in patients with LSS compared with that in normal people, especially in the multifidus.

Previous studies found that age, gender and BMI may influence the degeneration of paraspinal muscle (2, 18, 21). In order to increase the comparability, we used propensity score matching to minimize the differences in general conditions between the two groups. As the results showed in **Table 1**, there was no significant difference in general conditions, indicating the two groups were matched well.

The change rule of paraspinal muscle in patients with LSS was similar to that in normal people. From top to bottom of the spinal axis, the relative cross-sectional area of MF increased, while that of ES decreased. The FI and the rSI of MF and ES increased gradually. Another research measured the volume of paraspinal muscle in patients with LSS and found that the volume of paraspinal muscle was the largest at L3-4 level and gradually decreased toward the caudal end (22). They measured the volume of paraspinal muscle rather than parameters of both MF and ES, which was different with our study, but the change rule of paraspinal muscle was similar to our finding.

Recently many studies focused on the degeneration of paraspinal muscle in patients with chronic nonspecific low back pain and lumbar degenerative diseases. Ogon found that intracellular lipid content in multifidus muscle cells was higher in patients with chronic nonspecific low back pain than that in patients with LSS (7). Yagi found that the cross-sectional area of multiuse muscle was significantly smaller in patients with degenerative scoliosis combined with LSS than that in patients with single LSS (15). Liu found that FI in multifidus muscle at L5-S1 could be a predictor of functional improvement after surgery in patients with L4-5 single-segment LSS (14). But few studies investigated the differences of paraspinal muscle between patients with LSS and normal people.

By comparing the paraspinal muscle parameters between the two groups, the degeneration of paraspinal muscle was worse in patients with LSS than that in normal people. For parameters at L3 level, the rfCSA, FI, rSI of MF and rSI of ES were higher in patients group than those in normal group. Besides, the FI and rSI of MF at L4 level and the rSI of MF at L5 level were also higher in patients group. Moreover, the mean value of parameters from L3 to L5 level, such as rfCSA, FI, rSI of MF and rSI of ES were also significantly higher in patients group than those in normal group. Paraspinal muscle is important to maintain the spinal stability. The atrophy of paraspinal muscle led to the increase of fatty cross-sectional area and decrease of functional cross-sectional area, which was associated with the decrease in muscle strength (23), resulting in weakness in maintaining spinal stability. So we should pay attention to the degeneration of paraspinal muscle in LSS.

The degeneration of MF was significantly worse in patients than that in normal people, which may relate to the denervation of muscle fibers and differences in muscle stress. Yoshihara found that the denervation caused by compression of nerve root may lead to the degeneration of type I and type II fibers, causing structural changes of multifidus muscle (24). So nerve compression in patients with LSS may cause more degeneration of multifidus muscle.

In addition, the degeneration of MF was significantly different between the two groups, while there was no significant difference in degeneration of ES. While maintaining the spinal stability, the paraspinal muscle also bears the stress from the body or activities. The stress within physiological range is help to the exercise of muscle, but the overlord stress will cause muscle injury or degeneration. Previous studies reported that the MF bear more stress than the ES (25, 26), which may explain the degeneration of MF was worse rather than the ES. However, there also existed different conclusions. Lee measured the paraspinal muscle parameters of 650 patients from CT test and found that the atrophy of ES appeared earlier and more severe than the MF, which may relate to the difference of anatomical structure (27). Our conclusion was different with Lee’s, which may be associated with the difference of paraspinal muscle measurements and characteristics of population. In this study, we measured paraspinal muscle parameters from T2-weighted MRI, while Lee measured those from CT. All patients in this study were diagnosed as LSS, while the subject in Lee’ study was patient without spinal surgery, deformity and neuromuscular diseases. The inclusion criteria were broader than those in this study. The differences of paraspinal muscle degeneration need to be further explored.

In normal people, we found that the rfCSA and FI of MF, rfCSA and FI of ES and rSI of MF were significantly greater in females than those in males. In patients with lumbar disease, the FI of MF was also higher in females than that in males (18, 28). Compared to above results, there was no significant difference in paraspinal muscle parameter between different genders in patients with LSS, but the atrophy of paraspinal muscle was more severe in female than that in male. In addition, compared with normal people, the degeneration of multifidus muscle was higher in patients with lumbar spinal stenosis under different genders.

Age was an important factor for the degeneration of paraspinal muscle in patients with LSS and it was positively correlated with the rfCSA, FI and rSI of MF and ES, indicating that the degeneration of paraspinal muscle increased gradually with age. This result was consistent with previous studies (16, 18, 28, 29). We used the linear regression analysis to evaluate the relationship between age and FI of MF. The slope of fitted linear was higher in patients than that in normal people, indicating that with the increase of age, the degeneration of MF increased more significantly in patients than that in normal people. Shahidi analyzed the data of 199 patients who were between 18 and 80 years old. They found that the FI of MF and ES increased with age. They further compared paraspinal muscle parameters with the data of healthy people from Crawford’s study and found that the age-fat infiltration rate fitted line had a higher slope in female patients (18). Their results were consistent with this study. Except for age, weight and BMI were significantly correlated with the rtCSA of ES rather than the rtCSA of MF, suggesting that weight mainly affected the quantity of erector spinae.

In this study, propensity score matching was used to ensure the comparability between patients with lumbar spinal stenosis and normal people and reduce the influence of individual factors on the results. Paraspinal muscle parameters from L3 to L5 and the mean value of parameters were compared between the two groups and the differences in paraspinal muscle parameters were comprehensively analyzed. Patients with lumbar spinal stenosis are mainly middle-aged and elderly people, so this study focused on middle-aged and elderly people over 50 years old. However, there are some shortcomings in this study. Firstly, this study is a single-center study, which may have selection bias. Secondly, the number of people included in this study was small and the study with a larger sample size is needed to verify our results in the future.

## Conclusion

The change rules of paraspinal muscle in patients with lumbar spinal stenosis were similar to those in normal people. The degeneration of paraspinal muscle was more severe in patients with lumbar spinal stenosis than that in normal people, especially in multifidus. The degeneration of paraspinal muscle in patients with lumbar spinal stenosis was mainly related to age and the effect of age on atrophy of paraspinal muscle was greater than that of normal people.

## Data availability statement

The raw data supporting the conclusions of this article will be made available by the authors, without undue reservation.

## Ethics statement

The studies involving human participants were reviewed and approved by the Ethics Committee of Peking University Third Hospital. The patients/participants provided their written informed consent to participate in this study.

## Author contributions

WW conceived the project and analyzed the data. All authors contributed towards the interpretation and the collection of the data. All authors contributed to the article and approved the submitted version.

## Funding

This study was funded by Tianjin Key Medical Discipline (Specialty) Construction Project (TJYXZDXK-026A).

## Conflict of interest

The authors declare that the research was conducted in the absence of any commercial or financial relationships that could be construed as a potential conflict of interest.

## Publisher’s note

All claims expressed in this article are solely those of the authors and do not necessarily represent those of their affiliated organizations, or those of the publisher, the editors and the reviewers. Any product that may be evaluated in this article, or claim that may be made by its manufacturer, is not guaranteed or endorsed by the publisher.
